# Ultrasound-Mediated Drug Delivery: Sonoporation Mechanisms, Biophysics, and Critical Factors

**DOI:** 10.34133/2022/9807347

**Published:** 2022-01-29

**Authors:** Juan Tu, Alfred C. H. Yu

**Affiliations:** ^1^Key Laboratory of Modern Acoustics (MOE), Department of Physics, Collaborative Innovation Center of Advanced Microstructure, Nanjing University, Nanjing, China; ^2^Schlegel Research Institute for Aging, University of Waterloo, Waterloo, ON, Canada

## Abstract

Sonoporation, or the use of ultrasound in the presence of cavitation nuclei to induce plasma membrane perforation, is well considered as an emerging physical approach to facilitate the delivery of drugs and genes to living cells. Nevertheless, this emerging drug delivery paradigm has not yet reached widespread clinical use, because the efficiency of sonoporation is often deemed to be mediocre due to the lack of detailed understanding of the pertinent scientific mechanisms. Here, we summarize the current observational evidence available on the notion of sonoporation, and we discuss the prevailing understanding of the physical and biological processes related to sonoporation. To facilitate systematic understanding, we also present how the extent of sonoporation is dependent on a multitude of factors related to acoustic excitation parameters (ultrasound frequency, pressure, cavitation dose, exposure time), microbubble parameters (size, concentration, bubble-to-cell distance, shell composition), and cellular properties (cell type, cell cycle, biochemical contents). By adopting a science-backed approach to the realization of sonoporation, ultrasound-mediated drug delivery can be more controllably achieved to viably enhance drug uptake into living cells with high sonoporation efficiency. This drug delivery approach, when coupled with concurrent advances in ultrasound imaging, has potential to become an effective therapeutic paradigm.

## 1. Introduction

In the past several decades, the scientific community has witnessed great progress in realizing “smart drug delivery” as an advanced approach to deliver gene or drugs into specific locations of patients’ body with controlled dosage release, enhanced delivery efficiency, improved biocompatibility and easy accessibility [1–12]. On this topic, ultrasound-activated mechanical force has been regarded as one of the most promising strategies to realize spatiotemporally-controllable drug delivery to selected regions [2, 3, 6, 13–18]. This approach generally works by sending ultrasound extracorporeally to the treatment target to induce acoustic cavitation that is attributed to the dynamic activity of gas bodies. In turn, the interactions between cavitation bubbles and living cells would trigger the so-called “sonoporation,” which is characterized by the temporary disruption of cell membrane integrity, to facilitate the uptake of exogeneous gene/drug molecules into cells [19–28]. Note that sonoporation rarely happens in normal biological tissues and blood vessels, because gaseous cavitation nuclei seldom exist naturally within the human body. To facilitate the instigation of sonoporation, synthetic microbubbles (i.e., the same ones that are used as contrast agents in ultrasound imaging) are often introduced to the human body via intravenous injection into the circulation. These microbubbles usually comprise a gas-filled core (e.g., perfluorocarbon) encapsulated by a thin stabilization coating (e.g., lipid, albumin or polymer), with an average size between 1 and 8 *μ*m so that they are capable of passing through the pulmonary capillary bed [29–31]. It has been well accepted that the presence of synthetic microbubbles would effectively enhance acoustic energy absorption and lower the cavitation threshold, thereby amplifying the magnitude of cavitation-induced bioeffects [32–35]. Accordingly, various therapeutic applications have been developed based on both in vitro and in vivo gene/drug delivery studies [2, 14, 15, 31, 36–38], biomarker extraction [39], cancer treatment [36, 40–43], blood-brain barrier opening [44–49], neurostimulation [50], cardiovascular disease treatment [51, 52], and the treatment of chronic bacterial infection [53, 54].

Although the feasibility of leveraging sonoporation for drug delivery applications has been well demonstrated, the efficiency of this ultrasound-mediated therapeutic strategy has been a subject of concern [2]. In particular, data have shown a significant variation in the level of drug uptake that may be achieved without affecting cell viability [55]. As a result, there has been an increasing urge to better understand the fundamental science of sonoporation so that this process can be applied more rationally in therapeutics [5, 56] . Not only is there a need to more systematically study the range of factors that may influence the instigation of sonoporation but there is also a requisite to identify the multitude of biophysical responses and subcellular bioeffects that may be induced by sonoporation. Without such scientific knowledge, sonoporation may be applied in a brute manner that results in controversial therapeutic outcomes filled with confounding factors.

This article aims to consolidate the research community’s current understanding on the scientific foundations of sonoporation. It is our intent to provide an integrative overview on the existing body of observational evidence available on the notion of sonoporation, the prevailing understanding of the principal mechanisms involved, and the range of influencing factors and cellular responses that are related to this membrane perforation approach. In doing so, we intend to shed light on how future applications of sonoporation in therapeutics can be more rationally developed. Such rationalization effort will critically bolster the overall potential of ultrasound in becoming an effective theranostic modality that synergizes its imaging and therapeutic applicability.

## 2. Observational Evidence on Sonoporation

It has been two decades since sonoporation has been conceptualized as a membrane perforation approach. In 1999, Tachibana et al. used scanning electron microscopy (SEM) to reveal the existence of sonoporation sites on the membrane surface of HL-60 cells that were exposed to ultrasound in the presence of microbubbles [10]. After that, similar static photos of sonoporation sites have been reported by other groups using a variety of microscopy tools [57–64]. While these static images serve the purpose of confirming the presence of sonoporation sites, they inherently do not provide insight into the spatiotemporal dynamics of sonoporation sites. Such a lack of understanding on sonoporation dynamics has made it difficult for researchers to establish a working knowledge of the essential pore kinetics that should be taken into account when designing drug delivery applications.

One potent way of investigating the spatiotemporal dynamics of sonoporation is to perform live microscopy of the cell membrane with the help of membrane-specific fluorescent dyes. An example of such a sonoporation dynamic research platform is a live confocal microscopy setup with calibrated acoustic exposure conditions [65, 66]. Its key points of merit are that (i) individual, site-specific sonoporation can be controllably induced by ultrasound-triggered collapse of a single microbubble; (ii) the corresponding cellular dynamics can be tracked live over the entire course of sonoporation. Acoustically coupled confocal microscopy for sonoporation dynamic studies can offer a bold image contrast of the plasma membrane, whilst achieving sufficient temporal resolution (with real-time frame rates achievable) for tracking the dynamics of sonoporation sites during the initial growth phase and the recovery phase, which tend to happen on the order of seconds [65]. As such, this microscopy technique is potent in yielding new insights not obtainable with other techniques. In particular, it has enabled dynamic visualization of the plasma membrane, which is optically transparent and has not been imaged properly in optical imaging studies [64, 67–72]. Also, it can render the entire course of membrane perforation and recovery, thereby providing temporal insights of sonoporation sites not available in static photos of pores [57–64] or indirect monitoring of consequential events like exogenous marker uptake [73, 74]. Moreover, if combined with high-speed imaging system, similar live microscopy setup can also be adopted to realize indepth study on the dynamic process of acoustic droplet vaporization, as well as its sonoporation outcomes (e.g., membrane deformation, permeabilization, and blebbing response) on adjacent cells [75].

### 2.1. Direct Observations on Membrane Perforation and Recovery in Sonoporation

**Figure 1:**
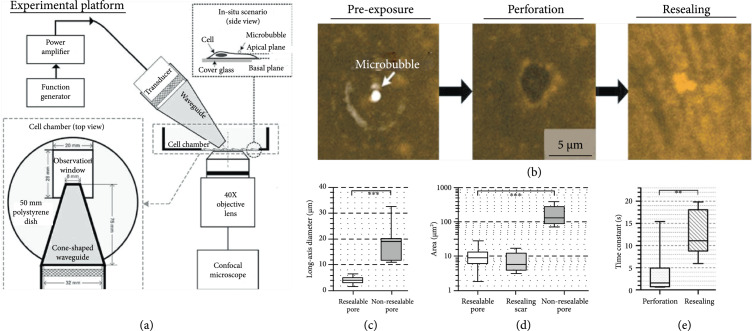
Membrane perforation and recovery dynamics in single-site sonoporation. (a) Diagram of the acoustically coupled confocal microscopy platform used for this investigation. (b) Representative sonoporation site images from perforation to recovery. (c, d) Temporal peak pore diameter and area for sonoporation sites that resealed successfully and unsuccessfully (N=7; ${}^{***}p<0.001$). (e) Characteristic time for perforation and recovery for the 7 successfully resealed sonoporation sites (${}^{**}p<0.01$), referenced to literature [65].

Figure 1 summarizes a series of membrane-level direct observations that are obtained with acoustically coupled live confocal microscopy [65]. These findings served well to epitomize the notion of sonoporation. For the first time, they have revealed how membrane perforation, and its subsequent resealing took place in a sonoporation episode. In that investigation, single-site sonoporation was achieved through delivering a single-shot ultrasound pulse (frequency: 1 MHz; pulse duration: 10 cycles; hydrophone calibrated peak negative pressure in situ: 0.85 MPa) to cavitate a single lipid-shelled microbubble (Targeson) adhered on the surface of anchored fetal fibroblast cell. Over this process, confocal microscopy of the plasma membrane (tagged using CellMask) was imaged in real-time. Perforation was observed to be synchronized with the instant of ultrasound pulsing.

The resealing process of sonoporation sites is a noteworthy dynamic event that has been revealed by live confocal microscopy. The study in Ref. [65] particularly found that the temporal peak area and the maximum long-axis diameter of resealable sonoporation sites were, respectively, less than 30 *μ*m^2^ and 7 *μ*m. Also, for these pores, their closure was generally completed within 1 min after sonoporation (with characteristic time constant below 20 s) [65]. In contrast, sonoporation sites were found to be unable to recover if their size was overly large (>100 *μ*m^2^). Same recovery failure happened if extracellular calcium ions (Ca^2+^) were chelated (note: Ca^2+^ influx is needed for wound repair initiation [76]).

**Figure 2:**
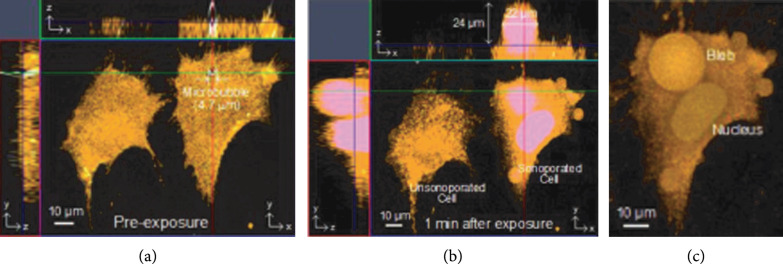
Bleb emerges at the sonoporation site. (a, b) 3D confocal imaging of plasma membrane (orange; maximum intensity projection shown on three planes). Two cells are shown: (left) an unsonoporated cell; (right) a cell sonoporated by ultrasound-triggered microbubble collapse. Sytox blue was used as sonoporation tracer (giving pink cytoplasmic fluorescence when merged with orange membrane). (c) Volumetric rendering of membrane bleb (top-down view), referenced to literature [78].

Beyond visualization of sonoporation sites, live confocal microscopy, which can acquire volumetric information about the membrane through a 3D scan, also revealed the formation of membrane blebs after sonoporation. Blebbing is recognized as an alternate repair strategy when passive reunion and patching are not effective [77]. Figure 2 shows that single-site sonoporation mediated by the collapse of a relatively large microbubble (4.7 *μ*m diameter) would lead to the generation of a membrane bleb at the puncturing site [78]. In this case example, 3D rendering of the plasma membrane was shown for two breast carcinoma cells: one that remained unsonoporated and another with sonoporation. For the sonoporated cell (confirmed by cytoplasmic presence of Sytox), a significant bleb had emerged at the preexposure microbubble position shortly after the onset of sonoporation (1 min), and its size was 24 *μ*m in apical height and 22 *μ*m in diameter. This feature was not observed in the unsonoporated cell.

### 2.2. Direct Observations of Subcellular Dynamics in Sonoporation

The impact of sonoporation extends beyond membrane-level disruptions. At a subcellular level, sonoporation has been found to disrupt the dynamics of the actin cytoskeleton [66]. After all, the actin network, as a subcellular scaffold, is interconnected with the plasma membrane [79]; so, concomitant disruption of actin during a sonoporation episode is not an unexpected event. Note that the actin disruption observations were acquired using a similar single-shot, single-microbubble experimental protocol as that described in the sonoporation site dynamic study [65], whereby the actin behavior (traced using CellLight Actin-GFP) during sonoporation was imaged in real-time. This investigation was conducted on breast carcinoma cells (ZR-75-30) that exhibit higher membrane deformability and hence a more fluidic actin scaffold [80]. Such property is known to protect cells from physical injury and favor recovery after a wounding event.

**Figure 3:**
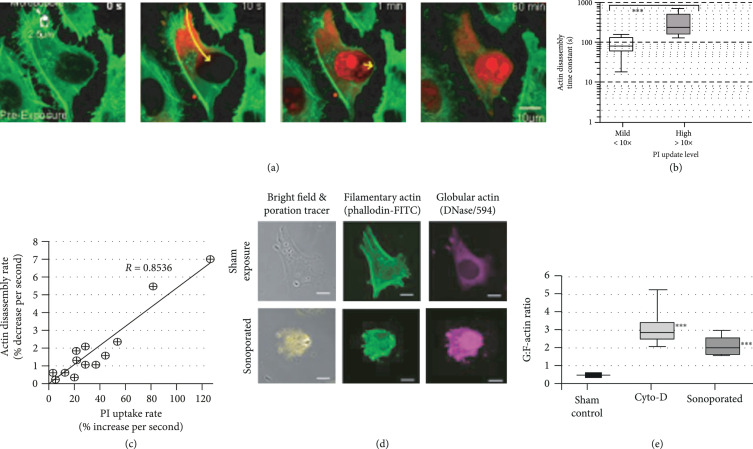
Single-site sonoporation would disrupt actin cytoskeleton. (a) Confocal images showing F-actin disassembly in a sonoporation episode (green: actin-GFP; red: propidium iodide). (b) For cells with higher tracer uptake, they have a larger actin disassembly time constant (N=8; ${}^{***}p<0.001$). (c) Actin disassembly rate is correlated with tracer uptake rate (N=16). (d) Fixed cell images at 2 h after onset of sonoporation, showing F-actin severance and G-actin increase. (e) G:F-actin imbalance in sonoporated cells is similar to Cyto-D drug-induced actin depolymerization (N=50; ${}^{***}p<0.001$ with respect to sham control), referenced to literature [66].

Figure 3 shows that, synchronous with membrane puncturing, an immediate rupturing of filamentary actin (F-actin) can be observed at the sonoporation site (Figure 3(a)). It is observed that the actin disassembly time constants for cells with mild PI uptake are generally of the order of tens seconds, while those for the cells with high PI uptake can reach over 100 s (Figure 3(b)). The pooled data also illustrates that, for the sonoporated cells, a positive correlation can be observed between their actin disassembly rate and PI uptake rate (Figure 3(c)). In addition, a domino effect can be observed in which the impact of sonoporation on actin was not limited to the initial disruption—further disassembly of the F-actin network was found to take place over the next 60 min, a time frame that was significantly longer than the membrane resealing time (on the order of tens of seconds [65]). The extent of F-actin disruption was more substantial in cells with higher uptake of sonoporation tracer. Also, a commensurate rise of globular actin (G-actin) was found as a consequence (Figure 3(d)), and in turn, the G:F-actin balance of the sonoporated cell was disrupted (Figure 3(e)). This latter finding shows that sonoporation may have long-term implications on the functional activeness of the cell’s actin machinery. It matches well with previous findings that showed perturbation of downstream cellular behavior after sonoporation [81, 82].

## 3. Physical and Biological Processes Related to Sonoporation

### 3.1. Physical Principles of Sonoporation

Beyond the acquisition of observational evidence on sonoporation, the research community has attempted to elucidate the mechanism of action involved in this membrane perforation approach. From a physic standpoint, the onset of sonoporation is well accepted to be closely related to microbubble dynamics. As mentioned already, microbubbles are suitable agents for sonoporation because of their cavitational interactions with ultrasound. Because of their gas core, microbubbles would alternate between expansion and shrinkage in response to the negative and positive phases of the incident ultrasound pulses [29, 83–85].

**Figure 4:**
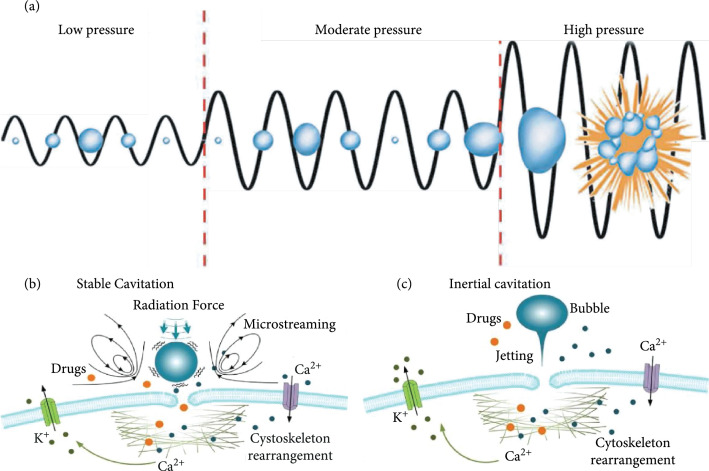
Schematic overview of major biophysical effects occurring during sonoporation generated by microbubbles. (a) A microbubble that is undergoing stable cavitation (SC), as triggered by low acoustic pressure, would exhibit symmetrical linear oscillations; in contrast, inertial cavitation (IC), which results in violent microbubble collapse and fragmentation, is excited by ultrasound exposures at even higher driving pressures. (b) A stably cavitation microbubble would generate volumetric oscillations to exert radiation force and microstreaming-induced shear forces. **(**c**)** An inertially cavitating microbubble would produce microjets. These physical forces can result in cell membrane permeabilization, cytoskeleton rearrangement, and transmembrane ion transport such as K^+^ efflux and Ca^2+^ influx.

It is well known that two types of cavitation dynamics may be exhibited by microbubbles in response to ultrasound excitation, viz., stable cavitation (SC) and inertial cavitation (IC). As illustrated in Figure 4(a), at very low acoustic pressures, microbubbles undergo SC that is characterized by symmetric linear oscillations. Their expansion and compression are inversely proportional to the local acoustic pressures [85–87]. At slightly higher ultrasound pressures (several tens of kilopascals), microbubbles undergoing SC would exhibit repeated small-amplitude asymmetric oscillations with a lengthened expansion phase [85–88]. If microbubbles reside in close vicinity of a cell (see Figure 4(b)), their oscillations would exert a cellular massage effect to disrupt plasma membrane integrity via a push-and-pull maneuver that corresponds to the expansion and compression phases of an oscillation cycle, as ultrafast optical microscopy has shown [22]. It has also been demonstrated that SC may generate radiation force that consequently presses microbubbles against the cell membrane and eventually break its integrity [25, 89–92]. Moreover, SC may induce fluid microstreaming that in turn generates shear stress on the cell membrane. This shear stress, which may be higher than several kilopascals, is regarded to be sufficiently strong to tear the cell membrane [25, 93, 94]. It may lead to various cellular impacts such as cytoskeleton rearrangements and nucleus contraction [23, 26, 65, 66, 92, 95–98].

In contrast to SC, microbubbles undergoing IC exhibit large-amplitude asymmetric microbubble oscillations, eventually resulting in violent microbubble collapse and fragmentation [59, 84, 99–102]. This type of cavitation is excited by ultrasound pressure levels higher than those used to induce SC (e.g., greater than several hundred kPa). During IC, shock waves and/or liquid jets are formed due to the asymmetrical microbubble implosion (see Figure 4(c)). If these phenomena arise adjacent to the cell membrane, they can puncture the cell surface to cause membrane perforation and cytoskeleton rupture and even permeabilize the endothelial membrane of blood vessels [18, 59, 60, 66, 67, 99, 101–105]. In general, IC can generate larger sonoporation sites than those generated by SC [59, 73, 106]. As discussed in Section 2.1, live confocal microscopy has shown that micron-sized sonoporation sites may be created [66], but submicron pore sizes may also be created as confirmed by voltage clamp measurements [107]. After the impact of acoustic cavitation has been delivered, the time required to reseal sonoporation sites may range from milliseconds to seconds for submicron pores, as indicated by an obvious decay of membrane permeabilization [65, 96, 101, 102, 108]. For larger sonoporation sites, membrane resealing is typically longer (tens of seconds), while pores that are excessively large may be irrepairable [65].

### 3.2. Biological Responses of Sonoporated Cells

Mechanical stress induced by sonoporation, whether it is induced via SC or IC, may lead to a variety of biological effects. First, as mentioned in Section 2.1, membrane blebbing has been acutely observed after the onset of sonoporation, and it is hypothesized to be a repair maneuver for sonoporation sites [78]. Another biological response that may be elicited is that reactive oxygen species (ROS) can accumulate within the cytoplasm following sonoporation [109–114], which can enhance cell membrane permeability and modulate the ion channels to stimulate Ca^2+^ influx [110, 115]. This biological response would concurrently lead to depolarization of the cell membrane potential [116]; subsequently, secondary factors such as the activation of voltage-gated ion channels may overcompensate such membrane potential disruption and lead to membrane hyperpolarization [117]. It has also been reported that the endocytosis process, which is helpful for the extracellular uptake of relatively large molecules [61, 118], could be activated by the mechanosensors sensitive to shear-force-induced membrane tension variations, ROS-mediated Ca^2+^ influx, and the hyperpolarization produced by K^+^ efflux.

Beyond the acute bioeffects of sonoporation, downstream repressive effects have been found in sonoporated cells. These downstream bioeffects include cell-cycle arrest, morphological repression [81], the induction of endoplasmic reticulum stress [82], suppression of clonogenicity [119], and ultimately apoptosis [120, 121]. The scale of biological response is seemingly heterogeneous between sonoporated cells depending on the extent of permeabilization, as flow cytometry data have shown [122, 123]. These downstream bioeffects would affect the viability of sonoporated cells, in addition to the potential of sonoporation to trigger immediate cell lysis [124]. Interestingly, similar biological impact of sonoporation has also been observed in plant cells whose cell wall is mechanically more rigid than mammalian cell membrane [125, 126].

## 4. Factors Affecting Sonoporation Dynamics and Bioeffects

### 4.1. Factors Related to Acoustics

Sonoporation-mediated drug delivery to cells is a multifaceted process with interplay between the level of acoustic energy, the characteristics of microbubbles or therapeutic agents, and the properties of targeted cells. The acoustic energy source can be adjusted by different driving parameters, as will be discussed in the following subsubsections.

#### 4.1.1. Ultrasound Frequency

The cavitation behavior of microbubbles at a certain frequency will highly depend on their size, as microbubble response will be much greater around their resonant radius [93]. Therefore, 1-MHz driving frequency is utilized because this frequency is in principle closer to the resonant frequency of commercialized ultrasound contrast agent microbubbles, whose diameters are normally between 1 and 3 *μ*m [26, 59, 95, 96, 101, 102, 106, 111, 112, 127–135]. However, in the microbubble solutions with relatively broad size distribution, the sonoporation efficiency would become stronger at lower driving frequency. For instance, Karshafian et al. investigated the sonoporation outcomes of KHT-C cells in suspensions with the presence of definity microbubbles ranging in size between 1 and 8 *μ*m, and the results showed that higher cell permeability and lower cell viability would be induced with 500 kHz ultrasound sonication than 2 MHz and 5 MHz exposures [136].

#### 4.1.2. Acoustic Pressure and Cavitation Dosage

Ultrasound pressure, particularly the peak negative pressure, is well considered as a determining factor for the size of sonoporation sites [127]. As demonstrated by a series of transmembrane current measurements, when the peak negative pressure is set to 0.12 or 0.3 MPa, the pore size is measured to range between 10 and 100 nm [73, 107]. In another SEM experiment where the peak negative pressure is set to 1.1 MPa, pores sizes of up to 1 *μ*m have been measured [62, 63]. Almost all published studies reported that an increase in acoustic pressure would result in enlarged membrane pores [59–61, 64, 65, 73, 119], as well as enhanced membrane permeability and transfection efficiency [59, 63, 102, 106, 124, 133, 134, 136–141]. However, these enhancements are commensurate with an increased loss of cell viability by (i) promoting immediate cell lysis and apoptosis or (ii) enhancing drug cytotoxicity [95, 106, 133, 136, 138, 139, 142].

**Figure 5:**
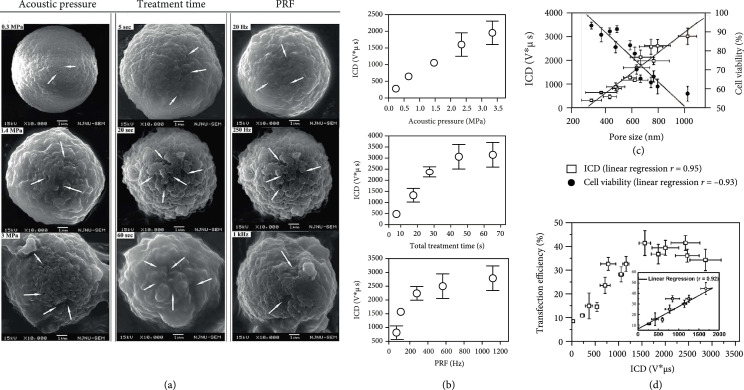
Correlation between sonoporation, IC dose (ICD), and gene transfection in MCF-7 cells. (a) SEM revealed that sonoporation sites are larger at higher ultrasound pressure, longer treatment time, and higher PRF (experiment was conducted with 1 MHz ultrasound). (b) The corresponding ICD is significantly enhanced as the three ultrasound parameters are increased. (c) The size of sonoporation sites is positively correlated with the measured ICD but is negatively correlated with cell viability. (d) Sonoporation-facilitated transfection of deoxyribonucleic acid (DNA) initially shows linear correlation with ICD, but this trend levels out at higher ICD values, referenced to literature [59].

Larger pores (hundreds nanometers to micrometers), which enable higher uptake of drugs with larger molecular weight [56, 59, 119, 143], are usually generated as a result of IC [22, 59, 64, 65, 73, 144]. On the other hand, relatively small pores (several tens to a few hundred nanometers) are often generated as a consequence of SC [60, 61]. Systemic SEM experiments (Figure 5(a)) have revealed that, when the peak negative pressure is increased from 0 to 3.0 MPa, the size of sonoporation sites is enlarged from about 150 nm to 1 *μ*m [59]. The corresponding ICD, as measured via passive cavitation detection, is found to show positive linear correlation with the peak negative pressure and the size of sonoporation sites [59] (Figures 5(b) and 5(c)). Note that the viability of sonoporated cells tends to be negatively correlated with ICD. As a result, gene transfection efficiency shows a complex correlation trend with ICD, in which there is an initial positive correlation but this is followed by saturation and even negative correlation at higher ICD values that tend to yield nonresealable pores (Figure 5(d)).

#### 4.1.3. Exposure Duration

Exposure duration is another important factor that may affect the extent of sonoporation. The reason is because the accumulated acoustic energy delivered to cells is after all equal to the product between the acoustic intensity and the total “on time” exposure duration, the latter of which can be varied by adjusting acoustic pulse length [124, 136, 145], PRF [59, 136, 146], duty cycle [136, 145–147], and total sonication time [59, 124, 134, 136, 146, 148]. A summary table (Table 1) is provided to list some typical articles discussing the major temporal factors that may affect sonoporation outcomes.

**Table 1:** Major temporal factors affecting sonoporation outcomes.

Impact factors		Sonoporation outcomes		
Category	Range	Transfection efficiency	Cell viability	Repairing rate
Total time↑	0.1-900 s	↑ [59, 125, 135, 137, 147, 149]	↓ [59, 125, 137, 149]	
Pulse length↑	4-1000 *μ*s	↑ [125, 137, 146]	↓ [125, 137, 146]	↓ [146]
Duty cycle↑	2%-90%	↑[137, 146, 147, 148]	↓[137, 146, 147, 148]	↓ [146]
PRF↑	10-3000 Hz	↑ [59, 137]	↓ [59, 137]	

Note: ↑ and ↓ represent the increase and decreased in corresponding categories.

Typically, the use of a shorter pulse length has been suggested to reduce cavitation-induced shear stress that is responsible for membrane pore generation [26]. In the case of single-pulse ultrasound exposure, pulse length is the same as the exposure time, which has been shown to positively correlate with transfection efficiency and negatively correlate with cell viability [136, 148]. Sonoporation may also be achieved efficiently using very short pulses (a few microseconds) if combined with high acoustic pressures [119, 149]. In general, increases in total exposure time and PRF would result in a higher ICD, enlarged pore size, and enhanced transfection efficiency, while reducing cell viability [59]. Nevertheless, the differences of transfection efficiency at different PRFs might be minor at fixed duty cycles [146].

### 4.2. Factors Related to Microbubble Properties

Microbubbles play a key role in sonoporation by providing cavitation nuclei [29, 150–155]. Therefore, it is not surprising that the extent of sonoporation may be affected by microbubble properties, such as microbubble size, concentration, and shell materials. The impact of these microbubble parameters on the extent of sonoporation will be discussed in the following.

#### 4.2.1. Bubble Size and Concentration

It is well known that the cavitation behavior of microbubbles is strongly dependent on their size, because stronger dynamic responses can be activated around their resonant radius [86, 156–158]. In regular medical ultrasound applications with a working frequency between 0.5 and 5 MHz, larger bubbles can normally induce stronger acoustic responses and enhanced membrane permeability than smaller bubbles [73, 132, 133]. Nevertheless, sometimes it is hard for microbubbles to penetrate the endothelial gaps (380-780 nm) within tumor blood vessels. Under such circumstance, nanobubbles with diameters of 300-700 nm are superior to microbubbles by achieving enhanced permeability and retention (EPR) effect in tumors [159, 160].

**Figure 6:**
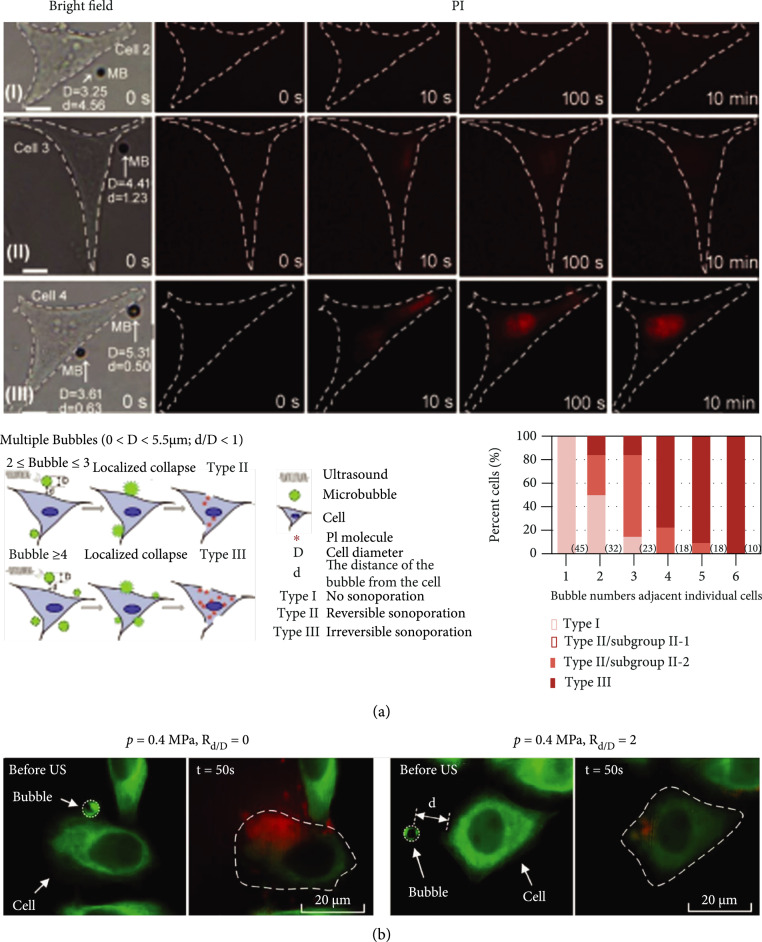
(a) The extent of sonoporation depends on bubble size and the number of bubbles adjacent to the cell. (I-III) Bright-field and propidium iodide (PI; model drug marker) fluorescence images of sonoporated cells at different times before and after a single 10 *μ*s, 1 MHz ultrasound pulse at 0.6 MPa peak negative pressure. When the bubble had a diameter (D) smaller than 5.5 *μ*m and the bubble-cell distance (d) was smaller than *D*, PI uptake (red fluorescence) shows positive correlation with bubble size and bubble number, referenced to literature [131]. (b) Real-time fluorescence observations on cellular responses induced by microbubble-mediated sonoporation at different d values. Higher PI uptake and significant cytoskeleton disassembly (green fluorescence) are observed when the ratio between d and D ($R_{d/D}$) was reduced from 2 to 0, referenced to literature [95].

The number of bubbles adjacent to the cell also can affect the extent of sonoporation. Fluorescent microscopy observations on single cells undergoing sonoporation have revealed that the extent of sonoporation tends to be less predictable if it is induced by large bubbles (diameter greater than 5.5 *μ*m) that exhibited translational movement over large distances [131]. The number of bubbles present near a cell also correlated positively with the extent of sonoporation, especially for smaller bubbles (with diameter smaller than 5.5 *μ*m) [131]. Moreover, sonoporation episodes triggered by localized collapse of fewer than three bubbles are generally reversible, whereas those triggered by the cavitation of four or more bubbles tend to be irreversible (Figure 6(a)). Generalizing these single-cell findings to a population of sonoporated cells, studies have found that an increase in microbubble concentration can effectively lower microbubble cavitation threshold [129, 153] and improve transfection efficiency [63, 134, 146, 161–164]; although, cell viability may be concomitantly compromised [146, 162, 164]. Yet, microbubble concentrations greater than 2% generally would not result in further increase in transfection efficiency [38, 161].

#### 4.2.2. Bubble-to-Cell Distance

Many studies have revealed that effective membrane perforation cannot happen unless the oscillating microbubbles get close enough to the cells. As such, the bubble-cell distance is another factor important for microbubble-mediated sonoporation. In general, sonoporation efficiency can be significantly improved with reduced bubble-cell distance [74, 101, 131, 138, 162]. At a single cell level, membrane perforation has been observed with 75% probability when the ratio between bubble-cell distance and microbubble diameter (*R*_D/d_) is 75% [165]. In such a scenario, IC-induced shock wave is generally regarded as an upstream course of action [101]. It is worth noting that, as shown via fluorescent microscopy (Figure 6(b)), a decrease in $R_{D/d}$ not only enhances membrane permeability but it also results in more severe cytoskeleton disassembly for HeLa cells [95]. These experimental observations are supported by numerical simulations, which suggested that the extent of cell membrane deformation is greater with higher acoustic pressure or reduced bubble-cell distance [95, 99, 162]. Note that, in order to shorten the bubble-cell distance as much as possible, targeted microbubbles may be fabricated by making use of specific ligands or antibodies to further enhance the drug delivery efficiency [74, 166].

#### 4.2.3. Bubble Shell

The shell of microbubbles is another factor that influences sonoporation dynamics [119, 140, 167–170]. Some studies have been performed to compare the drug delivery efficiency mediated by microbubbles that are encapsulated with different types of shells [55, 119, 171, 172]. For example, lipid-coated definity microbubbles are considered to yield a higher therapeutic ratio than protein-shelled Optison bubbles [119]. This observation may be explained from a chemistry standpoint in which microbubbles coated with lipid material are known to possess a thinner and more flexible shell, whereas a shell composed of polymer or protein is typically thicker and more rigid [153, 173, 174]. Consequently, for lipid-shelled microbubbles, studies have found that they would yield a lower cavitation threshold and a stronger dynamic cavitation behavior [86, 153]. These microbubbles are also easy to fragment into smaller “daughter” bubbles to introduce more cavitation nuclei and sustain cavitation activities for longer duration [171].

**Figure 7:**
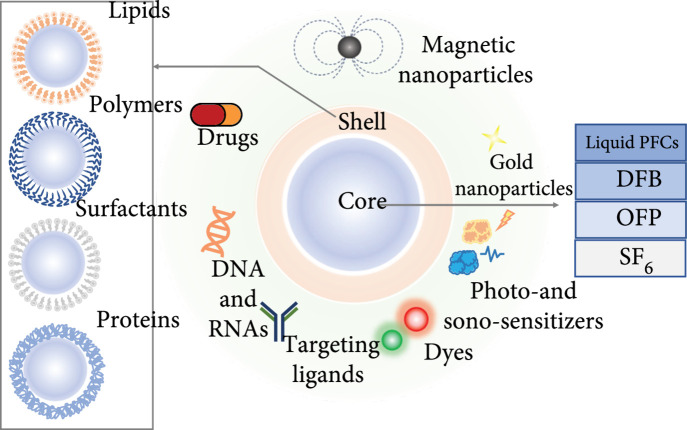
Biophysical properties and functional performance of contrast agent microbubbles can be modified by modifying the composition of microbubble shell and/or gas core.

In recent years, an increasing effort has been made to modify the shell properties of microbubbles, so as to improve their diagnostic and therapeutic performances (Figure 7). Examples include the fabrication of hollow or caged biodegradable microcapsules [170, 175–177] and integrating MRI/CT-sensitive nanoparticles (e.g., targeting ligands and drugs, gold nanoparticles, magnetic nanoparticles, photo- and sonosensitizers, and dyes) with microbubbles [133, 178–183]. In some studies, the impact of super paramagnetic iron oxide (SPIO) concentration on the properties of microbubbles has been characterized, including their shell stiffness, acoustic scattering response, and ICD, as well as relevant thermal effect [133]. It is found that (see Figure 7), with a higher SPIO concentration, larger and more flexible SPIO-albumin-shelled microbubbles may be fabricated to achieve stronger acoustic scattering, greater ICD, and higher temperature elevation, thereby benefitting their applications in drug and gene delivery and HIFU treatment [133, 181, 184, 185]. Peng et al. reported that caged microbubbles could be fabricated with varying shell elasticity and porosity to adjust their resonance frequency and control their cavitation modes [170]. Generally, microbubbles with porous and more compressible shells should possess lower resonance frequency and exhibit robust SC behaviors, while nonporous microbubbles would quickly transitioned to IC mode as the driving pressure was raised. A few studies have also demonstrated that perfluorocarbon gas-filled microbubbles are more efficient at inducing sonoporation than air-filled ones [186, 187], although the impact of gas core should be less significant than other microbubble parameters.

### 4.3. Factors Related to Cellular Properties

Since sonoporation after all occurs in living cells, it is reasonable for sonoporation efficiency to be dependent on cellular characteristics. As a simple example, cell density would inherently affect the level of hemolysis observed in red blood cells [155, 188, 189], because this parameter fundamentally affects the bubble-to-cell distance [161], whose impact has been discussed in Section 4.2.2. Aside from cell density, plasma membrane properties are known to vary between different cell types and different cell cycle phases, while extracellular conditions may also influence a cell’s response to sonoporation. Thus, variations are expected to arise in the extent of sonoporation induced with a given set of acoustic and microbubble parameters.

#### 4.3.1. Cell-Type Variations

Different cell types are known to exhibit different responses to sonoporation [122]. For instance, variations in the bioeffects observed in sonoporated cells have been found across different tumor cell types including breast tumor (MCF-7), liver tumor (Bel7402), ovarian tumor (A2780), and thyroid tumor (ARO) [134]. In turn, various levels of transfection and postsonoporation cell viability have been observed depending on the cell line used in the investigation [23, 130, 135, 142, 190]. The duration of sonoporation has also been reported to significantly differ between cell types [130].

#### 4.3.2. Cell Cycle Dependence

A few studies have yielded initial insight on the potential dependence of sonoporation impact on cell cycle phase. It has been observed that sonoporated cells in the $G_{2}/M$ phase (i.e. approaching cell division) have a higher transfection efficiency than those in $G_{0}/G_{1}$ or S phases [129]. Another study has shown that, for S-phase cells that are undergoing DNA synthesis, they have exhibited a higher level of model drug uptake in direct response to sonoporation, while their actin cytoskeleton have disassembled at a faster rate immediately after sonoporation [96]. Such observations may be mechanistically linked to different levels of microstreaming-induced shear stress exerted on cells in various cell-cycle phases whose membrane elasticity is known to differ [96, 191].

#### 4.3.3. Biochemical Factors

Contents in the extracellular fluid space may influence how a cell responds to a sonoporation episode. For instance, extracellular Ca^2+^ is known to be important in sonoporation site repair [76, 108, 142, 148], as the absence of this ion in the extracellular space would critically prevent the poration site from initiating repair [65, 76]. In contrast, the addition of synthetic nanoparticles to the extracellular space may modulate gene transfection efficiency. Specifically, it has been shown that, by adding polyethylenimine (PEI) into the extracellular fluid, this cationic polymer could effectively prolong the expression duration of genes transferred into cells via sonoporation [192]. An increase in the ratio between nitrogen and phosphate in the PEI polymer also would significantly enhance transfection efficiency [193].

Another factor that may affect the sonoporation efficiency is the culture environment. In order to achieve better reproducibility of biological effects resulted from microbubble IC activity, most microbubble-mediated sonoporation studies have been performed within the whole volume of medium mixed with suspended cells. However, stronger targeted microbubble attachment and more violent bubble oscillation were observed for cells cultrured on the rigid substrate, and higher pressure might be necessary to generate sonoporation pores on the membrane of cells cultured on the soft substrate [194]. To better understand the safety and efficiency of in vivo sonoporation, some attempts were also made to investigate sonoporation outcomes using monolayer cell samples to mimic in vivo tissue environment [195, 196].

## 5. Perspectives, Future Directions, and Conclusions

Although dedicated efforts have been made to unravel the essential science in sonoporation, most contemporary studies are generally centered upon either upstream physics or downstream bioeffects. Many specific mechanisms still need to be elucidated. For example, even though the concept of sonoporation has been originally conceived in the 1990s, its recovery mechanisms remain to be an obscurely described process as of today. Also, while acoustic cavitation is known to bring about mechanical impact of the cell membrane during sonoporation, the mechanosensitive signaling pathways involved in the process are still ill-defined and have lacked dedicated investigations. Even more, to push promising studies on sonoporation-mediated therapy closer to clinical applications, more considerations need to be highlighted for both clinical safety and therapy efficacy. It is well known that ultrasound contrast agent microbubbles are so sensitivity to acoustical exposures so that even the usage of diagnostic ultrasound may result in capillary rupture [197]. The violent collapse of cavitation bubbles may induce extremely high strain that can even achieve histotripsy [198], while causing undesired damage to surrounding normal tissues. Thus, there are urgent demands for advanced developments of more efficient visualization guidance technique, standardized metrology of cavitation dosage, and real-time feedback control technology based on intraoperative quantity-effectiveness evaluation.

In summary, future research still needs to be performed to fill these knowledge and technology gaps. The discovery of new biophysical mechanisms related to sonoporation would play an important role in establishing necessary knowledge for rationalizing the use of sonoporation in biomedicine. In the context of drug and gene delivery, such effort would serve well to enhance the efficiency of drug uptake by the treatment target, thereby improving the treatment outcome. Recognizing that ultrasound can be concurrently used for imaging purposes, rational realization of ultrasound-mediated drug delivery will significantly bolster endeavors that seek to develop ultrasound into an effective theranostic paradigm for biomedical applications.
